# Effect of gamma irradiation on the uptake, translocation, and phytotoxicity of lead and cadmium in a soil–barley (*Hordeum vulgare L.*) system

**DOI:** 10.3389/fpls.2025.1729964

**Published:** 2025-11-25

**Authors:** Xiaojie Wang, Baozhen Hao, Jingli Ma, Junfu Wang

**Affiliations:** School of Biological Engineering, Xinxiang University, Xinxiang, Henan, China

**Keywords:** gamma irradiation, barley, Cd–Pb contamination, Cd and Pb uptake, transportation and accumulation

## Abstract

**Aims:**

This study investigated how gamma irradiation affects the plant growth and the migration of cadmium (Cd) and lead (Pb) within the soil-barley system.

**Results:**

Our results demonstrated that gamma irradiation increased root length and root-shoot ratio compared with 0 Gy, as well as increased spike length, spikelet number, and spikelet grain weight. Furthermore, 50–150 Gy gamma radiation decreased Cd and Pb contents in grains compared to 0 Gy, Moreover, gamma radiation increased root bioconcentration factor for Cd and Pb (R-BCF_Cd_ and R-BCF_Pb,_ except for 120 Gy in Cd), while the BCF_Cd_ of the barley stem, leaf, and grain all decreased (except for 35 Gy in the leaf), Meanwhile, all gamma radiation treatments decreased translocation factor values from root to stem-leaf for Cd and Pb (R-S+L-TF_Cd_ and R-S+L-TF_Pb_) at maturity, with a differential response observed in translocation factor values from stem-leaf to grain for Cd (S+L-G-TF_Cd_). In addition, Pearson correlation analysis showed that the variability of Cd content in the grain was significantly and positively correlated with stem BCF_Cd_ (S-BCF_Cd_), grain BCF_Cd_ (G-BCF_Cd_), and R-S+L-TF_Cd_. Pb content in the grain was significantly and positively correlated with grain BCF_Pb_ (G-BCF_Pb_) and stem-leaf to grain for Pb (S+L-G-TF_Pb_).

**Conclusion:**

Our results suggest 50–150 Gy irradiation reduces heavy metal content in grains, likely by modulating physiological responses and the plant’s heavy metal transport pathways. This study offers a novel approach to low-cost pre-sowing seed treatment for mitigating grain metal contamination.

## 1 Introduction

Heavy metals are among the most widespread soil and environmental pollutants (Zwolak et al., 2019; Elik et al., 2022; Zhang et al., 2022), with Cd and Pb receiving the most concern due to their high persistence and toxicity (Wang et al., 2023; Yang et al., 2023). These two heavy metals are not only readily soluble in water but are also easily absorbed by plants, posing a serious threat to the environment (Duan et al., 2020; Peng et al., 2022; Zhang et al., 2022). Among the coexisting pollutants in contaminated farmland, the contents and mobility of Cd and Pb are significantly higher than those of other heavy metals (Li et al., 2020). The high mobility of these metals accelerates their transfer from soil to crops, leading to accumulation in different plant tissues, and when their levels exceed certain standards, they present substantial risks. Cd and Pb can cause toxic effects on crops, such as inhibiting normal cell division, reducing leaf photosynthetic efficiency, inducing lipid peroxidation of cell membranes, and suppressing antioxidant enzyme activity, which collectively hinder healthy plant growth and reduce yield (Rizwan et al., 2017; Martin et al., 2023; Zhao et al., 2024). Furthermore, by entry into the human body via the food chain, Cd and Pb can lead to kidney damage and reduced bone density, posing a severe threat to both human health and the environment (Soisungwan et al., 2020). Therefore, altering the ability of crops to absorb and transfer Cd and Pb in contaminated agricultural fields, as well as reducing their concentrations in grains, is essential for safeguarding human health.

Gamma irradiation has been widely applied as an improvement technique in various plant species given its easy availability and strong penetration ability (Prasad and Kumar, 2024; Şen et al., 2024; Neethu et al., 2022). The effects of gamma rays on plant growth and development are diverse, ranging from stimulatory to inhibitory responses (Jan et al., 2012; Caplin and Willey, 2018; Zhao et al., 2020), depending on factors such as radiation dose, plant species, age, genome organization, physiology, and morphology (Amirikhah et al., 2021; Gudkov et al., 2019). Numerous studies have demonstrated that gamma irradiation can improve various growth parameters, including germination, growth, vigor, and yield (Wani et al., 2019; Safoora et al., 2022; Santanu et al., 2022; Allayarova et al., 2023). In addition, low-dose gamma irradiation has been shown to enhance plant tolerance to abiotic stress (Zhang et al., 2016). However, the mechanisms by which gamma irradiation regulates Pb and Cd uptake, translocation, and bioaccumulation in different tissues of barley plants under Cd and Pb stress remain unclear.

Hence, this study aimed to assess the impact of gamma irradiation on (i) the growth parameters of barley throughout its developmental stages; (ii) the availability, enrichment, and transfer mechanisms of Cd and Pb (root–shoot–grain); and (iii) the yield traits of barley grown under Cd- and Pb-contaminated conditions. Overall, this study provides valuable insights for farmers and policymakers on effective strategies to manage Cd- and Pb-contaminated soils and to reduce metal uptake in crops, particularly barley.

## 2 Materials and methods

### 2.1 Soil preparation

Cd- and Pb-contaminated soil was collected from the 0–20 cm layer of a field (35°36’N, 113°85’E) in areas of Xinxiang City, Henan Province, China, located near battery production sites. All soil samples were air-dried, and gravel and biological residues were manually removed. The soil was then passed through a 2-mm sieve for further use and analysis. The basic characteristics and heavy metal contents of the soil were as follows: the soil was alkaline, with a pH of 7.31; total nitrogen was 43.51 mg/L; total phosphorus was 29.97 mg/L; the Pb content was 60.0 mg/kg; and the Cd content was 17.3 mg/kg.

### 2.2 Gamma radiation treatment

Uniform, healthy barley seeds were exposed to different doses of gamma radiation, including 35, 50, 75, 90, 120, and 150 Gy, using a ^60^Co gamma source at a dose rate of 6.25 Gy/h at the Institute of Isotopes, Academy of Sciences, Henan. Control seeds that were not irradiated were labeled as 0 Gy.

### 2.3 Pot experiment and sampling

**Figure 1:**
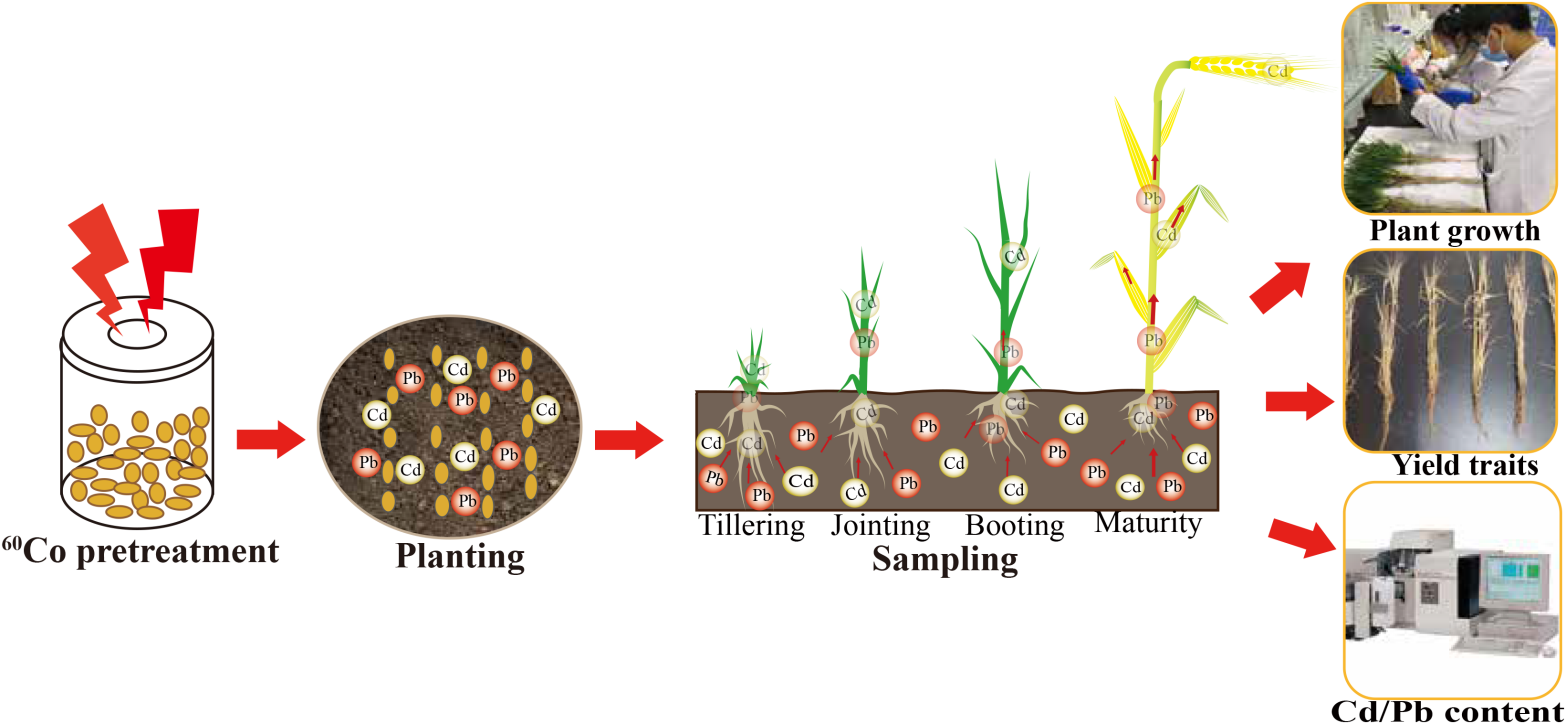
Schematic diagram of the experimental procedure of this study, including amendment preparation, cultivation, sampling, and parameter measurements.

The experimental process is shown in **Figure 1**. The pot experiment was conducted at Xinxiang University. The experimental design consisted of seven treatments: 0, 35, 50, 75, 90, 120, and 150 Gy. The pots, made of polyethylene, had an inner diameter of 35 cm and a height of 27 cm, with each containing 10 kg of soil. As a base fertilizer, 2.07 g/pot urea, 8 g/pot superphosphate, and 2.16 g/pot potassium sulfate were applied. Each treatment was replicated three times. There were 20 barley seeds that were sown per pot, and seedlings were thinned to 10 plants per pot after germination.

Plant samples were collected during the tillering, jointing, booting and maturity stages. Plants were separated into different organs according to their developmental stage, and tissue samples were placed in paper bags with plant parts loosened and separated. Samples were dried at 70 °C to a constant weight, weighed to determine dry weight, and subsequently sieved to < 1 mm for further analysis.

Soil samples were collected at the same growth stages. The collected soil samples were processed by removing stones, animal, and plant residues, gently pressing with a wooden rod, and then sieving through a 2-mm nylon mesh to remove larger gravel above 2 mm. The soil was further ground and passed through a 2-mm nylon sieve to a 100-mesh nylon sieve (0.149 mm aperture) for subsequent analysis.

### 2.4 Plant and soil analysis

#### 2.4.1 Agronomic traits analysis

Plant heights, root lengths, and root-shoot ratio from barley plants across different treatments were measured during the tillering, jointing, booting, and maturity stages. Soil and fly ash adhering to the roots were thoroughly removed by washing thoroughly under tap water, followed by rinsing with deionized water. Growth indicators were then determined. At the maturity stage, spike length, grain number per spike, grain weight per spike were measured.

#### 2.4.2 Physio-chemical property analysis

The pH was measured in a 1:2.5 soil-to-water suspension using a pH meter (Mettler Toledo FE20, Switzerland). Total nitrogen and total phosphorus were determined following the methods described in the Physical Chemical Analysis of soils.

#### 2.4.3 Heavy metal analysis

Tissue samples (0.1000 g) were transferred into polyethylene tubes and treated with 5 mL of a mixed acid solution (HNO_3_:HClO_4_ = 4:1). The tubes were covered with a lid, left overnight, and then digested, and the concentrations of Pb and Cd were analyzed. Soil samples (0.2000 g) were digested using a HNO_3_–HF–H_2_O_2_ (4:2:1) mixture, and the total contents of Cd and Pb were measured using an atomic absorption spectrometer.

### 2.5 Statistical analysis

All experiments were conducted in triplicate. One-way analysis of variance (ANOVA) was used to compare means among different treatments. Differences between individual means were evaluated using Duncan’s multiple range test in SPSS 20.0, with *p* < 0.05 considered statistically significant. Pearson correlation analysis was calculated using the R function “chart.Correlation” to assess the correlation relationships between Cd/Pb content and heavy metal accumulation, transportation. *, *p* < 0.05; **, *p* < 0.01; ***, *p* < 0.001. The *post hoc* test used for the estimation of the significance level is the Duncan test.

The following indicators were calculated based on established Equations 1–4: the translocation factor (TF) and bioconcentration factor (BCF) were determined using the equations described in previous studies (Zong et al., 2021; Yang et al., 2023; Chen et al., 2024).

(1) $$\text{T}{\text{F}}_{\text{R}-\text{A}}=\;{\text{C}}_{\text{aerial part}}/\;{\text{C}}_{\text{root}}$$

(2) $$\text{T}{\text{F}}_{\text{R}-\text{S}+\text{L}}=\;{\text{C}}_{\text{stem}-\text{leaf}}/\;{\text{C}}_{\text{root}}$$

(3) $$\text{T}{\text{F}}_{\text{S}+\text{L}-\text{G}}=\;{\text{C}}_{\text{grain}}/\;{\text{C}}_{\text{stem}-\text{leaf}}$$

(4) $$\text{BCF }=\;{\text{C}}_{\text{plant}}/\;{\text{C}}_{\text{soil}}$$

where TF_R-A,_ TF_R-S+L_ and TF_S+L-G_ are the Cd or Pb translocation factor values from root to aerial part, root to stem–leaf and stem–leaf to grain system, respectively. BCF is the Cd or Pb bioconcentration factor values from soil to barley. C_root,_ C_stem-leaf,_ C_grain,_ C _plant,_ and C _soil_ are the concentrations of Cd or Pb in the root, stem–leaf, grain, root–aerial part- stem–leaf–grain, and soil, respectively.

## 3 Results

### 3.1 Effects of gamma irradiation on the growth of barley under Cd and Pb stress at different growth stages

**Figure 2:**
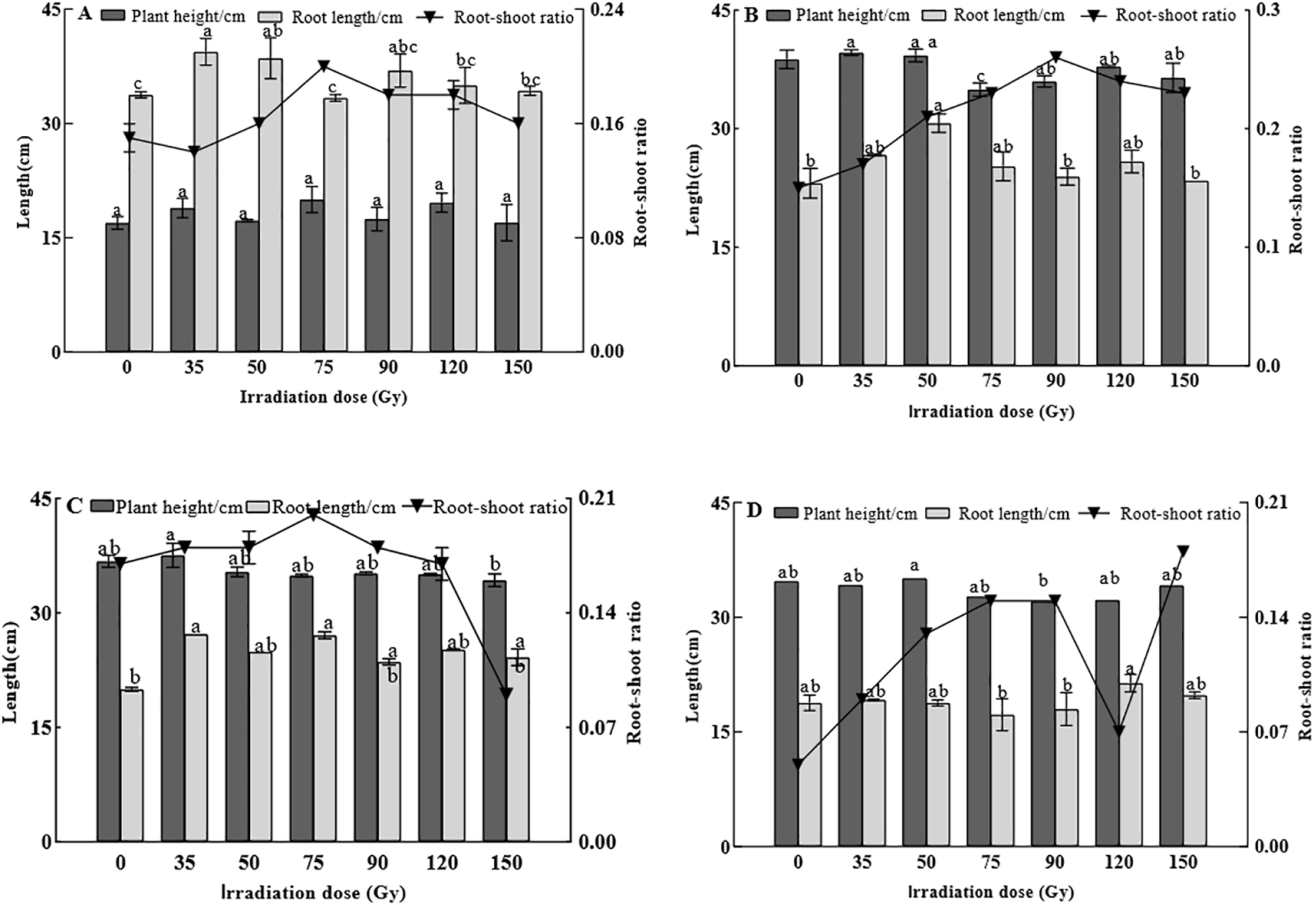
Effect of gamma irradiation on plant height, root length, and root-shoot ratio of barley. **(A–D)** show the parameters in tillering, jointing, booting, and maturity stages, respectively. The *post hoc* test used for the estimation of the significance level is the Duncan test, different small letters above the same column indicate a significant difference between treatments (*p* < 0.05).

To assess the level of enhanced tolerance to heavy metal stress under different doses of gamma irradiation, we measured plant height, root length, and root-shoot ratio at four key developmental stages. The results are shown in **Figures 2A–D**, During the tillering stage (**Figure 2A**), plant height increased under gamma irradiation doses of 35–150 Gy, with the maximum increase observed at 75 Gy. For root length, the longest roots were observed at 35 Gy (39.40 cm), while the shortest roots occurred at 75 Gy (33.37 cm). Regarding the root-shoot ratio, doses of 50–150 Gy led to an increase in this parameter in barley. During the jointing stage (**Figure 2B**), plant height under 35 Gy and 50 Gy treatments was higher than that of the 0 Gy control, while treatments of 75–150 Gy resulted in lower plant height. Root length under all irradiation treatments (35–150 Gy) was greater than that of 0 Gy, with 50 Gy showing a significant increase compared to the control. For the root-shoot ratio, all irradiation treatments led to higher values compared with 0 Gy. During the booting stage (**Figure 2C**), plant height was highest under the 35 Gy treatment, whereas the lowest height was observed under the 150 Gy treatment. For root length, compared with 0 Gy, all gamma irradiation treatments with 35, 50, 75, 90, 120, and 150 Gy resulted in increases of 36.18, 24.44, 35.33, 17.99, 26.09, and 20.99%, respectively. The root-shoot ratio was highest at 75 Gy and lowest at 150 Gy. During the maturity stage (**Figure 2D**), the tallest plants were observed under the 50 Gy treatment. Root length in barley under 75 and 90 Gy was lower than that for 0 Gy. The root-shoot ratio under all of the treatments (35–150 Gy) was higher than that of the control. In summary, although plant height is inhibited at higher doses, root growth (length) is significantly promoted across a much wider range of doses (35–150 Gy), especially during the jointing and booting stages. This indicates that the root system is either more resistant to the stress or is actively stimulated by it as a survival strategy. Under all of the irradiation doses (35–150 Gy), the root-shoot ratio consistently showed a systematic upward trend. This indicates a fundamental physiological shift in resource partitioning induced by gamma irradiation, thus serving as a crucial mechanism to mitigate stress and improve overall survival under challenging environmental conditions.

### 3.2 Effects of gamma irradiation on heavy metal absorption, enrichment and transport in barley at different growth periods

#### 3.2.1 Differences in heavy metal Cd content in different parts of barley

**Figure 3:**
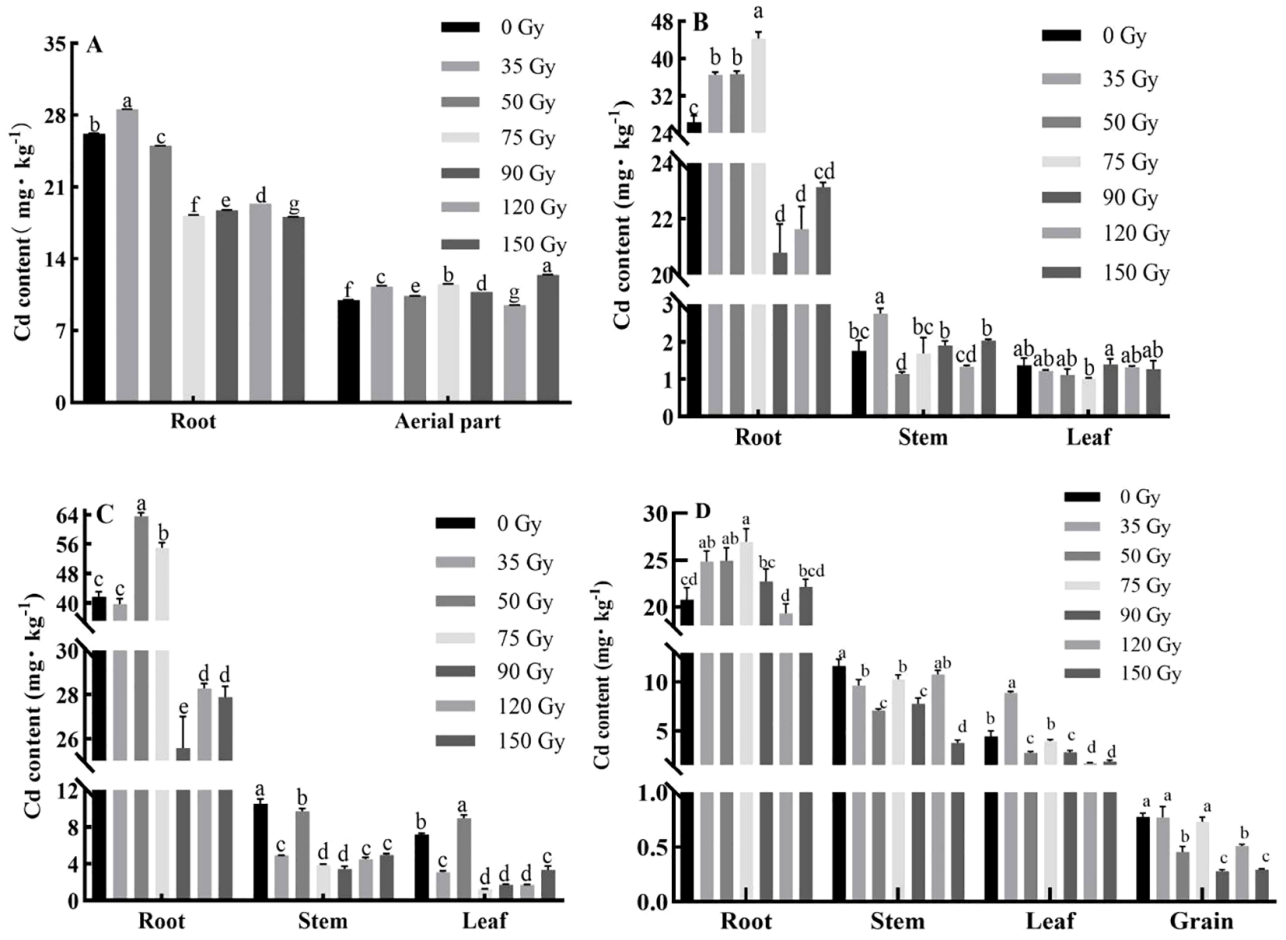
Effect of gamma irradiation on the Cd content in different parts of barley. **(A–D)** show the Cd content in different parts in tillering, jointing, booting, and maturity stages, respectively. The *post hoc* test used for the estimation of the significance level is the Duncan test, different small letters above the same column indicate a significant difference between treatments (*p* < 0.05).

In order to investigate how gamma irradiation influences Cd uptake and distribution in barley, we analyzed Cd content in different plant parts. At the tillering stage (**Figure 3A**), compared with 0 Gy, Cd content in roots increased by 9.06% under 35 Gy but decreased (4.48–31.01%) at higher doses (50–150 Gy). Cd content in aerial parts generally increased across treatments except at 120 Gy. During the jointing stage (**Figure 3B**), compared with 0 Gy, Cd content in roots increased (38.45–67.80%) under 35–75 Gy but declined (12.41–21.21%) under 90–150 Gy. Cd content in stems decreased under 50, 75, and 120 Gy, while Cd content in the leaves decreased under all of the doses except 90 Gy. During the booting stage (**Figure 3C**), compared with 0 Gy, Cd content in roots increased significantly under 50 and 75 Gy (by 52.64% and 31.99%, respectively), but decreased by 4.62–38.60% under other doses. Cd contents in stems and leaves were generally reduced across irradiation treatments, except for leaves at 50 Gy. During the maturity stage (**Figure 3D**), Cd content in barley roots, stems, leaves and grains were 19.33–26.98, 3.80–11.65, and 1.71–8.89, and 0.28–0.78 mg/kg. Compared with 0 Gy, Cd content in roots increased under all of the irradiation doses (12.41–21.21%, except 120 Gy) but decreased in stems (all of the doses), leaves (all doses except 35 Gy), and grains (all of the doses). Significant grain Cd reduction (34.62–64.00%) was achieved at 50, 90, 120, and 150 Gy.

#### 3.2.2 Differences in heavy metal Pb content in different parts of barley

**Figure 4:**
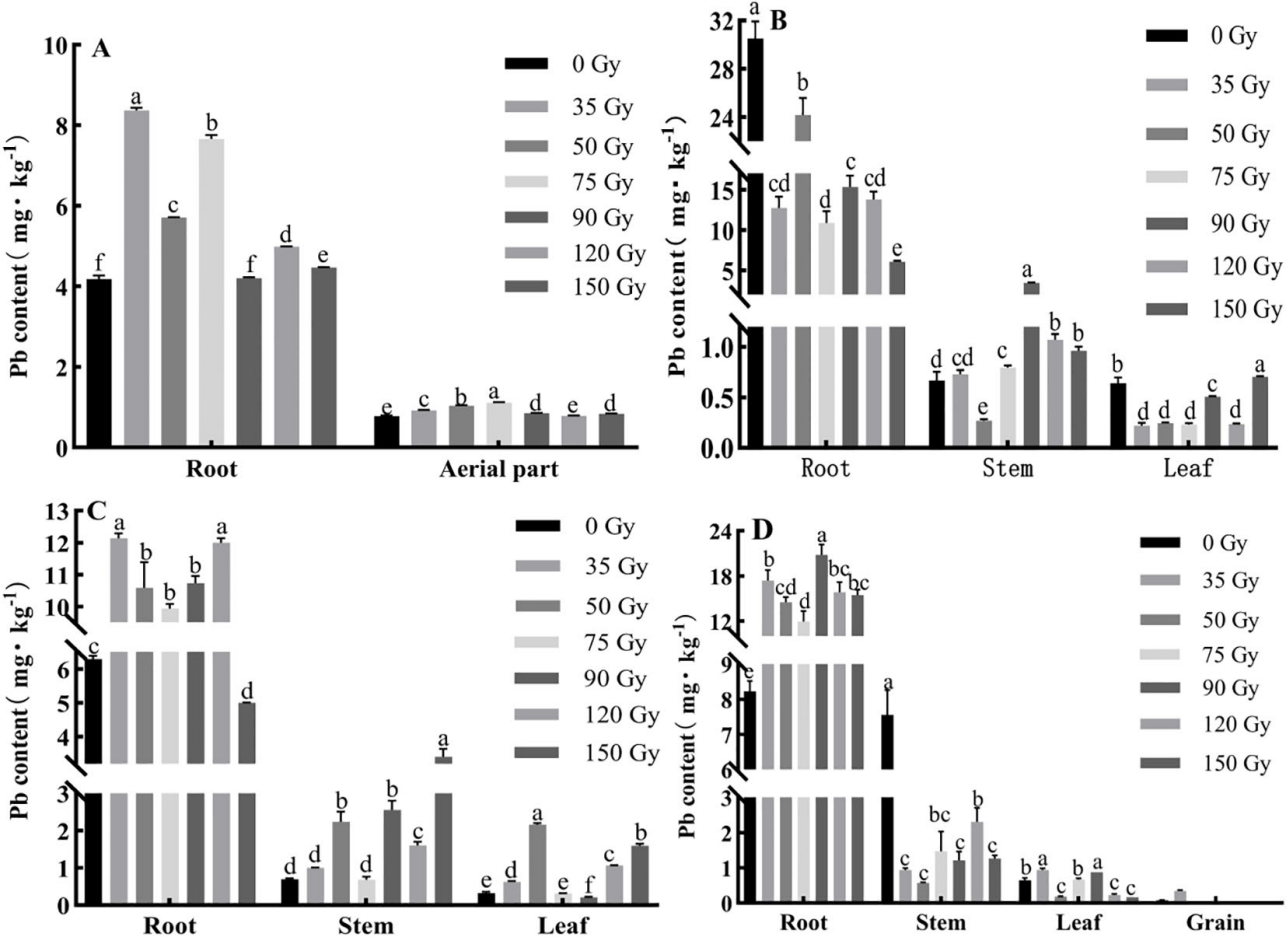
Effect of gamma irradiation on Pb content in different parts of barley. **(A–D)** show the Pb content in different parts in tillering, jointing, booting, and maturity stages, respectively. The *post hoc* test used for the estimation of the significance level is the Duncan test, different small letters above the same column indicate a significant difference between treatments (*p* < 0.05).

In order to determine how gamma irradiation affects the absorption and distribution of Pb in barley, we measured Pb content in its various organs. As shown in **Figure 4**, Pb content in different parts of barley varied under different gamma irradiation treatments and growth stages. During the tillering stage (**Figure 4A**), all of the doses increased root Pb content (0.42–99.51%), and aerial part Pb content (8.06–43.00%) compared with 0 Gy. During the jointing stage (**Figure 4B**), root Pb content decreased (20.74–80.04%) under all of the doses; stem Pb content increased (all except 50 Gy); leaf Pb content decreased (all doses except 150 Gy) relative to 0 Gy. During the booting stage (**Figure 4C**), Pb content in barley roots, stems, and leaves were 5.00–12.14, 0.69–3.40, and 0.21–2.15 mg/kg, respectively. The Pb content in the roots increased under all irradiation treatments except 150 Gy, with an increase range of 58.11–93.12% compared with 0 Gy. Similarly, the Pb content in the stems increased under all treatments except 75 Gy, and Pb content in leaves increased under all treatments except 75 and 90 Gy. During the maturity stage (**Figure 4D**), the Pb contents in barley roots, stems, and leaves were 8.23–20.80, 0.57–7.56, and 0.16–0.94 mg/kg, respectively. All irradiation treatments increased the Pb content in barley roots compared with the control, with 90 Gy having the most significant effect, enhancing root Pb content by more than 1.1-fold compared with the control. In contrast, Pb content in stems decreased under all irradiation treatments relative to the control. The Pb content in the leaves increased under 35, 75, and 90 Gy treatments compared with the control. In the case of grain Pb content, all treatments except 35 Gy had lower contents than the limit value stipulated in GB 2762-2022.

#### 3.2.3 Differences in BCF_Cd_ in different parts of barley

**Figure 5:**
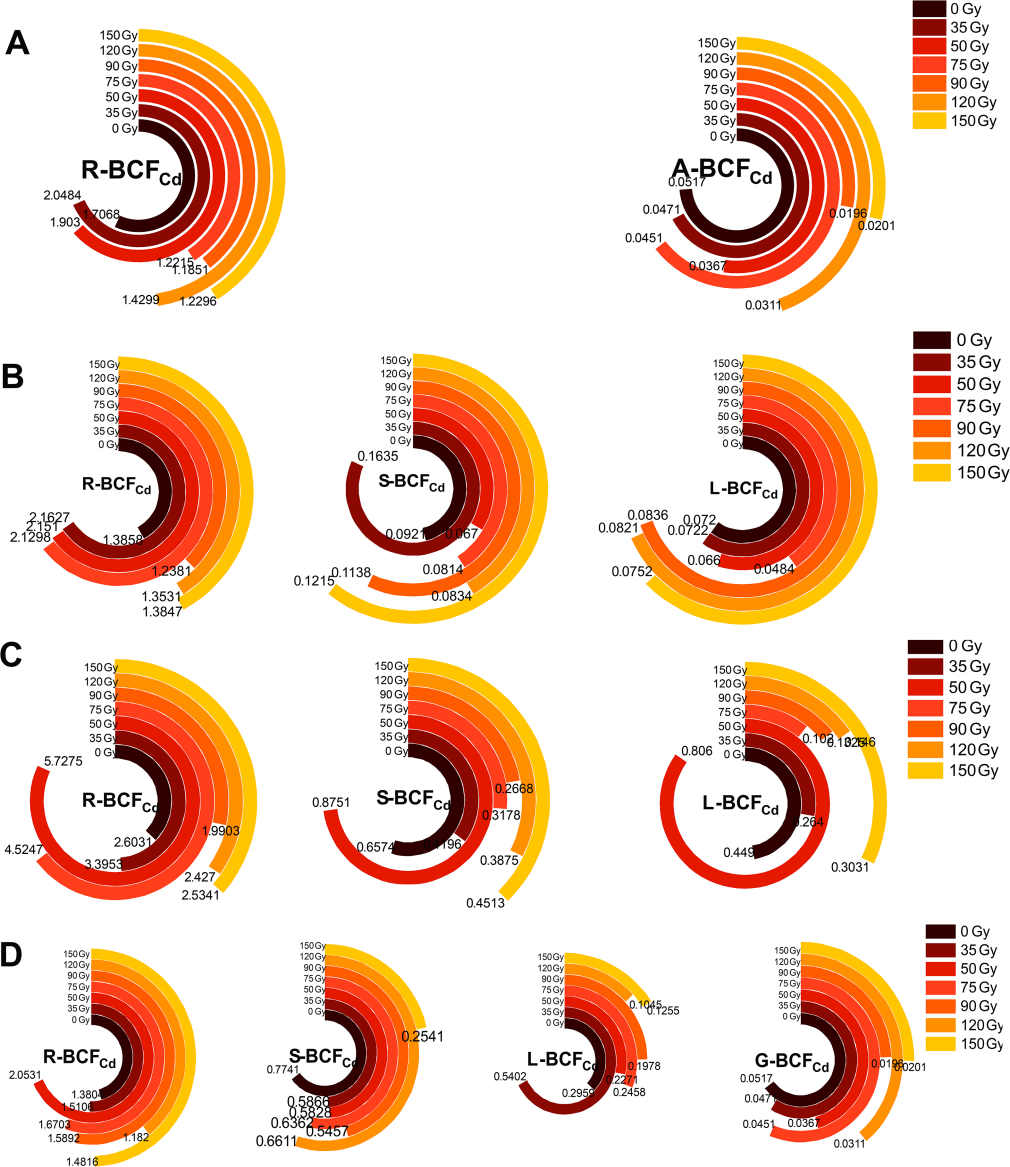
Effects of gamma irradiation on BCF_Cd_ in barley. **(A–D)** show the BCF_Cd_ in different parts in tillering, jointing, booting, and maturity stages, respectively. R-BCF_Cd_, root Cd bioconcentration factor; A-BCF_Cd_, aerial part Cd bioconcentration factor; S-BCF_Cd_, stem Cd bioconcentration factor; L-BCF_Cd_, leaf Cd bioconcentration factor; G-BCF_Cd_, Grain Cd bioconcentration factor.

In order to understand the mechanisms in metal distribution, we evaluated the plant’s efficiency in absorbing metals from soil and translocating them between organs. As shown in **Figure 5**, the BCF_Cd_ for different parts of barley varied under different gamma irradiation treatments and growth stages. With progression of growth, R-BCF_Cd_ initially increased and then decreased (**Figures 5A-D**), reaching a maximum during the booting stage, with a range of 1.99 to 5.73 for different treatments. Treatments of 35, 50, and 75 Gy increased R-BCF_Cd_ compared with that under 0 Gy, with the maximum increase observed at 50 Gy, which was 1.2 times higher than that of the control. The R-BCF_Cd_ remained consistently higher than that of aerial parts, while A- BCF_Cd_ remained below 1. During the maturity stage, BCF_Cd_ values for barley stems, leaves, and grains were 0.25–0.77, 0.10–0.54, and 0.02–0.05, respectively. All irradiation treatments decreased the Cd enrichment coefficient in the stems, leaves, and grains compared with that of the control (except for 50 Gy in L-BCF_Cd_).

#### 3.2.4 Differences in BCF_Pb_ in different parts of barley

**Figure 6:**
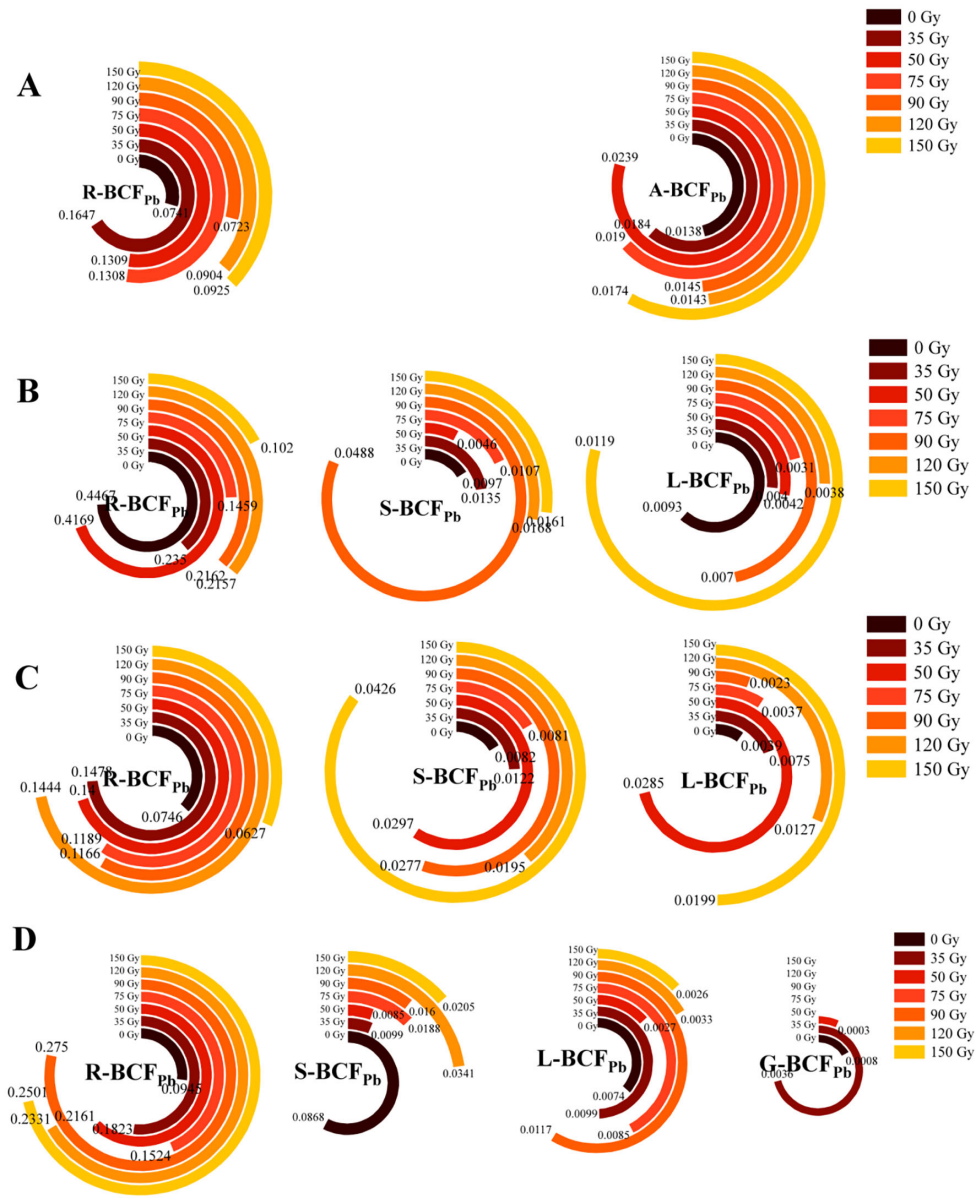
Effects of gamma irradiation on BCF_Pb_ in barley. **(A–D)** show the BCF_Pb_ in different parts in tillering, jointing, booting, and maturity stages, respectively. R-BCF_Pb_, root Pb bioconcentration factor; A-BCF_Pb_, aerial part Pb bioconcentration factor; S-BCF_Pb_, stem Pb bioconcentration factor; L-BCF_Pb_, leaf Pb bioconcentration factor; G-BCF_Pb_, Grain Pb bioconcentration factor.

As shown in **Figure 6**, the BCF_Pb_ of different parts of barley varied under different gamma irradiation treatments and growth stages. Over time, the R-BCF_Pb_ initially increased and then decreased (**Figures 6A-D**), reaching a maximum during the jointing stage and ranging from 0.10 to 0.45 across treatments. Among them, all of the irradiation treatments decreased the R-BCF_Pb_ and L-BCF_Pb_ compared with 0 Gy, (except for 150 Gy in leaves), whereas all treatments (except 50 Gy) increased S-BCF_Pb_. The BCF_Pb_ of all barley parts remained below 1, with R- BCF_Pb_ consistently higher than in the aerial parts. During the maturity stage, the BCF_Pb_ values of barley roots, stems, leaves, and grains were 0.09–0.27, 0.01–0.08, 0.0026–0.0099, and 0.0000–0.0036, respectively. Compared with 0 Gy, all irradiation treatments increased R-BCF_Pb,_ while decreasing S-BCF_Pb._ Treatments of 50, 120, and 150 Gy decreased L-BCF_Pb._ For the G-BCF_Pb,_ all irradiation treatments (except 35 Gy) resulted in lower values, with the treatments of 75–150 Gy showing values equivalent to 0 Gy.

#### 3.2.5 Differences in TF_Cd_ in different parts of barley

**Figure 7:**
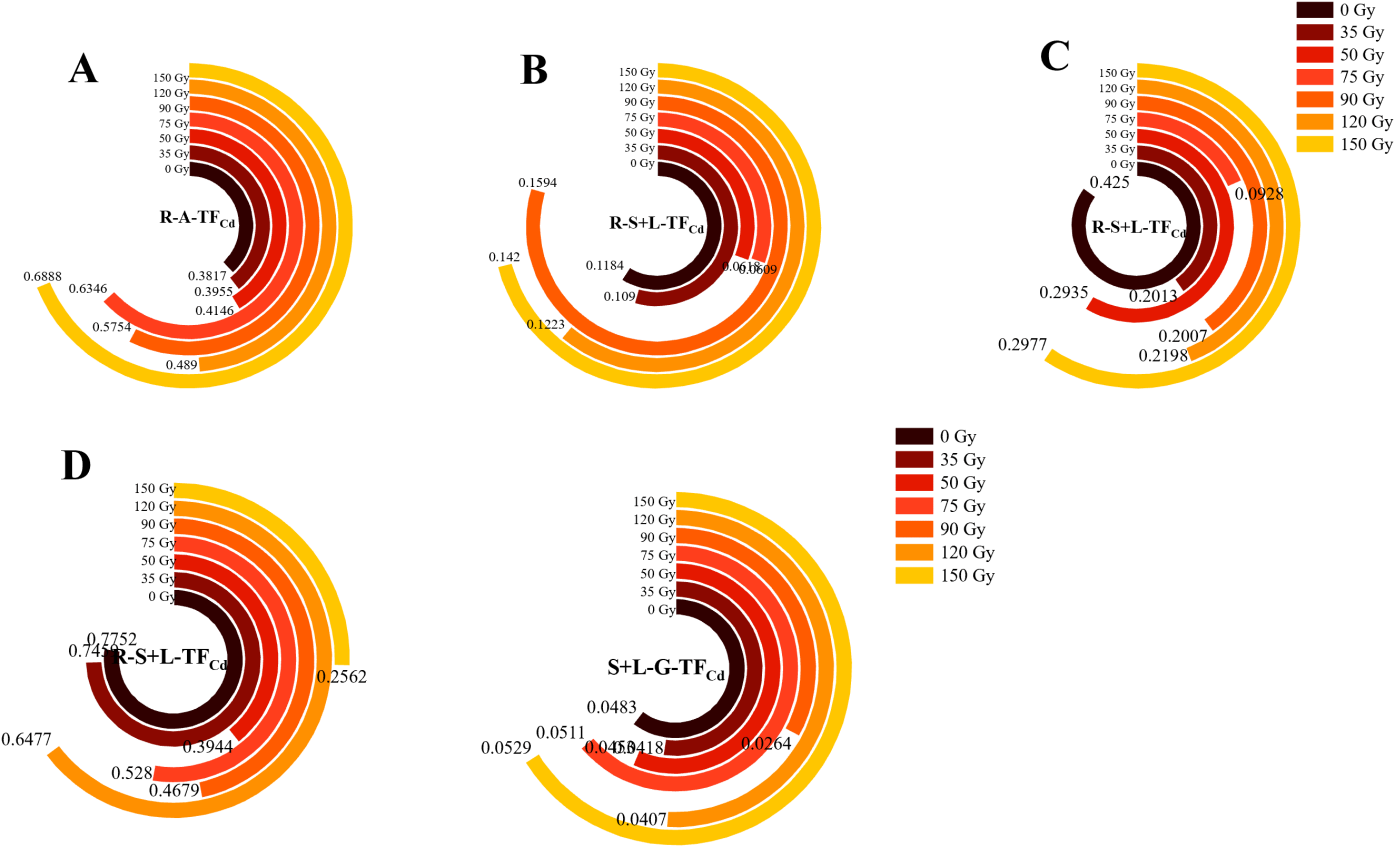
Effects of gamma irradiation on TF_Cd_ in barley. **(A–D)** show the TF_Cd_ in different parts in tillering, jointing, booting, and maturity stages, respectively. R-A-TF_Cd_, Cd translocation factor from root to aerial part; R-S+L-TF_Cd_,Cd translocation factor from root to stem-leaf; S+L-G-TF_Cd_, Cd translocation factor from stem-leaf to grain.

To compare the migration capacity of Cd in different parts and growth stages of barley, the coefficient of transport was used to evaluate transport from root to stem–leaf and from stem–leaf to grain. As shown in **Figure 7**, the TF_Cd_ varied in the different barley parts, with root to stem–leaf values in the ranges of 0.38–0.69, 0.07–0.16, 0.09–0.43, and 0.26–0.78 during the tillering, jointing, booting, and maturity stages, respectively (**Figures 7A-D**). During the tillering stage, all irradiation treatments increased R-A-TF_Cd_ by 3.61–80.46% compared with 0 Gy (**Figure 7A**). In contrast, during the booting and maturity stages, all irradiation treatments decreased these values by 29.96–78.17% and 0.04–66.94%, respectively. S+L-G-TF_Cd_ ranged from 0.026 to 0.053. Treatments of 35, 50, 90, and 120 Gy decreased this compared with 0 Gy, with reductions ranging from 0.06 to 45.48%. The 90-Gy treatment showed the most pronounced reduction. In contrast, treatments of 75 and 150 Gy increased S+L-G-TF_Cd_ by 0.06–0.10%. R-S+L-TF_Cd_ values were much larger than S+L-G-TF_Cd_, indicating that Cd is readily absorbed by barley roots and transported to the stems and leaves, but its transfer from stems and leaves to grains is limited.

#### 3.2.6 Differences in TF_Pb_ in different parts of barley

**Figure 8:**
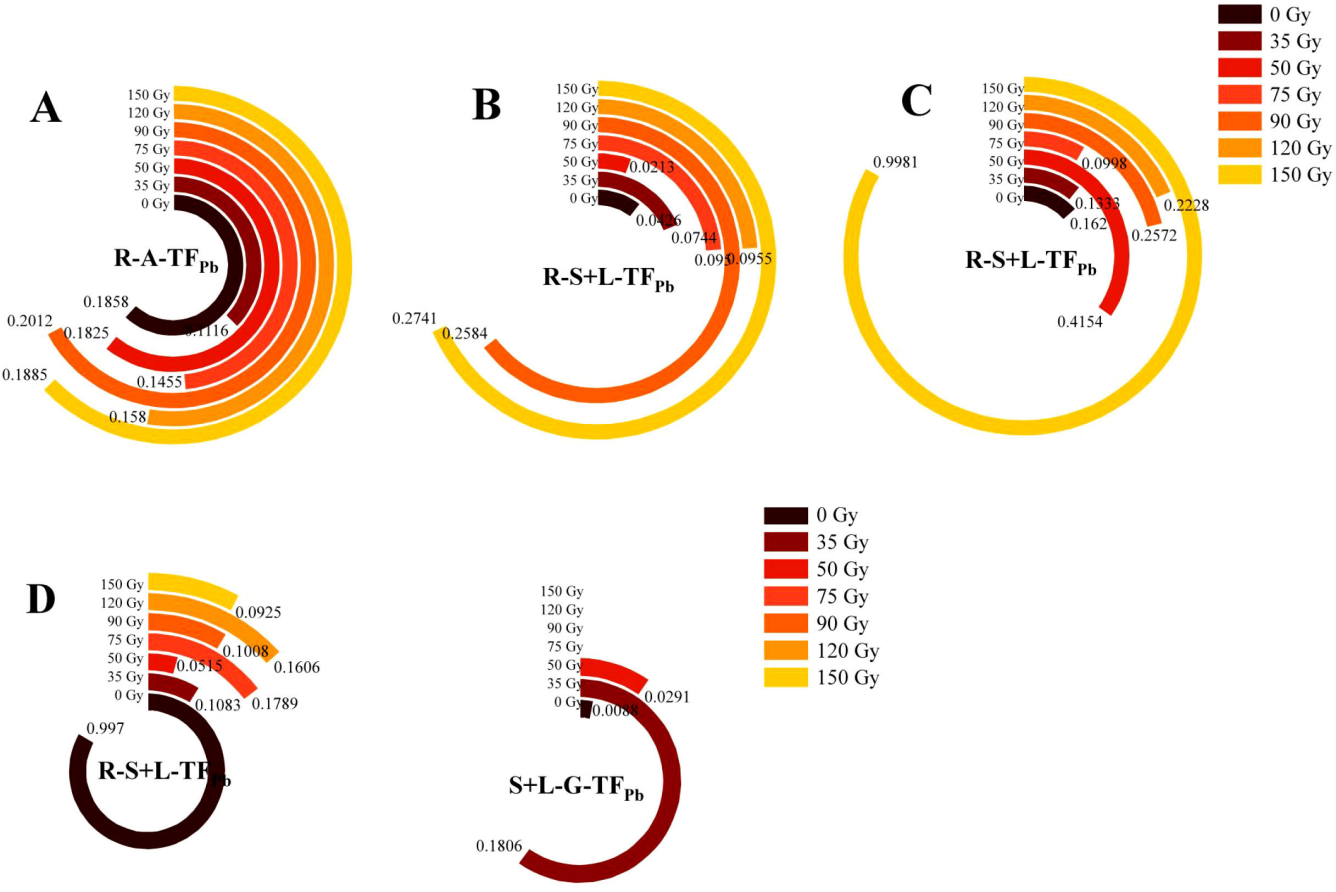
Effects of gamma irradiation on TF_Pb_ in barley. **(A–D)** show the TF_Pb_ in different parts in tillering, jointing, booting, and maturity stages, respectively. R-A-TF_Pb_, Pb translocation factor from root to aerial part; R-S+L-TF_Pb_, Pb translocation factor from root to stem-leaf; S+L-G-TF_Pb_, Pb translocation factor from stem-leaf to grain.

As shown in **Figure 8**, TF_Pb_ varied among the different parts of barley, with R-A-TF_Pb,_ R-S+L-TF_Pb_ values in the ranges of 0.11–0.20, 0.02–0.27, 0.01–1.00, and 0.05–1.00 during the tillering, jointing, booting, and maturity stages, respectively (**Figures 8A–D**). During the tillering stage, treatments of 90 and 150 Gy slightly increased R-A-TF_Pb_ by 0.01–0.08% compared with 0 Gy. However, treatments of 35, 50, 75, and 120 Gy decreased R-A-TF_Pb_ by 0.02–40.00%. In the jointing stage, all irradiation treatments except 50 Gy increased R-S+L-TF_Pb_, with 90 and 150 Gy significantly enhancing it by over 5.1-fold, and 75 and 120 Gy increasing it by over 1.2-fold compared with 0 Gy. During the booting stage, treatments of 50, 90, 120, and 150 Gy increased R-S+L-TF_Pb_ by 156.40, 58.76, 37.54, and 516.02%, compared with 0 Gy, whereas 35 and 75 Gy treatments decreased R-S+L-TF_Pb_ by 17.71–38.43%. During the maturity stage, all irradiation treatments reduced R-S+L-TF_Pb_ by 82.05–94.83% compared with 0 Gy, with 50 Gy showing the most pronounced reduction. S+L-G-TF_Pb_ ranged from 0.0000 to 0.1806, with 35 Gy resulting in the highest increase compared with 0 Gy, while S+L-G-TF_Pb_ in the 75–150 Gy treatments was measured as 0. These results are consistent with the Pb content observed in barley grains.

### 3.3 Effects of gamma irradiation on production-related indicators

The yield-related parameters for barley, including spike length, spikelet number, and spikelet grain weight, were measured to assess the effects of gamma irradiation on growth under heavy metal stress. As shown in **Table 1**, spike length ranged from 2.92 to 4.64 cm under different treatments. Treatments of 35, 50, 75, 90, 120, and 150 Gy increased the spike length by 14.34, 19.98, 47.54, 58.67, 38.64, and 40.70%, respectively. Similarly, gamma irradiation significantly increased the spikelet number in barley plants under heavy metal stress compared with the control. Specifically, treatments of 35, 50, 75, 90, 120, and 150 Gy increased spikelet number by 33.33, 44.44, 55.56, 55.56, 111.11, and 66.67%, respectively. Spikelet grain weight was also considerably increased upon gamma irradiation, except at 75 Gy upon heavy metal stress. The 35, 50, 90, 120, and 150 Gy treatments increased the spikelet grain weight by 38.10, 93.65, 115.87, 137.46, and 82.22%, respectively. In summary, the 35–150-Gy treatments increased spike length, grain number, and barley weight compared to the control.

**Table 1:** Effects of gamma irradiation on yield components of barley under Cd and Pb stress.

Gamma irradiation dose (Gy)	Spike length (cm)	Grain number per spike	Grain weight per spike (g)
0	2.92 d	9 d	0.39 d
35	3.34 cd	12 c	0.54 c
50	3.51 c	13 bc	0.76 b
75	4.31 ab	14 bc	0.43 cd
90	4.64 a	14 bc	0.85 ab
120	4.05 b	19 a	0.94 a
150	4.11 b	15 b	0.72 b

Different lowercase letters in the same column indicate significant differences among treatments (*p* < 0.05).

### 3.4 Correlation analysis

**Figure 9:**
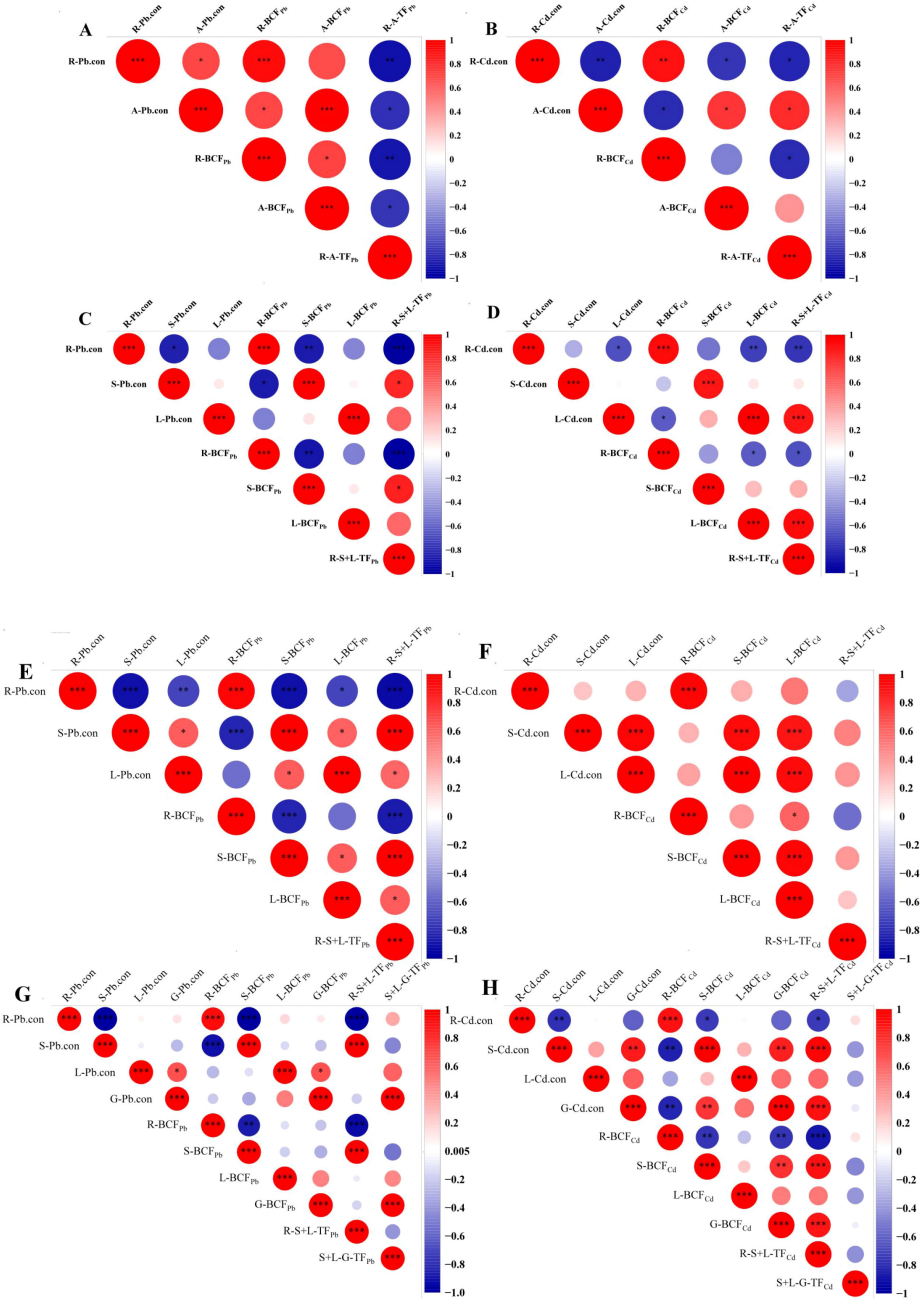
Correlation between heavy metal accumulation, transport, and heavy metal content in different parts of barley. **(A–H)** show the correlation in different parts in tillering, jointing, booting, and maturity stages, respectively. R-Cd.con, root Cd content; A-Cd.con, aerial part Cd content; S-Cd.con, stem Cd content; L-Cd.con, leaf Cd content; G-Cd.con, grain Cd content; R-Pb.con, root Pb content; A-Pb.con, aerial part Pb content; S-Pb.con, stem Pb content; L-Pb.con, leaf Pb content; G-Pb.con, grain Pb content; R-BCF_Cd_, root Cd bioconcentration factor; A-BCF_Cd_, aerial part Cd bioconcentration factor; S-BCF_Cd_, stem Cd bioconcentration factor; L-BCF_Cd_, leaf Cd bioconcentration factor; G-BCF_Cd_, grain Cd bioconcentration factor; R-BCF_Pb_, root Pb bioconcentration factor; A-BCF_Pb_, aerial part Pb bioconcentration factor; S-BCF_Pb_, stem Pb bioconcentration factor; L-BCF_Pb_, leaf Pb bioconcentration factor; G-BCF_Pb_, grain Pb bioconcentration factor; R-A-TF_Cd_, Cd translocation factor from root to aerial part; R-S+L-TF_Cd_,Cd translocation factor from root to stem-leaf; S+L-G-TF_Cd_, Cd translocation factor from stem-leaf to grain; R-A-TF_Pb_, Pb translocation factor from root to aerial part; R-S+L-TF_Pb_, Pb translocation factor from root to stem-leaf; S+L-G-TF_Pb_, Pb translocation factor from stem-leaf to grain.*p < 0.05; **p < 0.01; ***p < 0.001. The *post hoc* test used for the estimation of the significance level is the Duncan test.

The correlation between Cd/Pb content in plant tissues (root, aerial part, stem, leaf and grain) and heavy metal accumulation and transport under different treatments were analyzed. As shown in **Figure 9**, during the tillering stage, R-BCF_Pb_ exhibited a highly significant positive correlation with Pb content in different barley tissues. Similar results were observed for A-BCF_Pb_ and A-Pb.con. In contrast, R-A-TF_Pb_ showed a highly significant negative correlation with R-Pb.con. For Cd, R-BCF_Cd_ was highly positively correlated with R-Cd.con, and A-BCF_Cd_ was highly positively correlated with A-Cd.con. Additionally, R-A-TF_Cd_ showed a highly significant positive correlation with A-Cd.con, but a highly significant negative correlation with R-Cd.con (**Figure 9A**). During the jointing and booting stages, the correlation varied among the different parts of barley (**Figures 9B, C**). During the maturity stage, R-BCF_Pb_ showed a highly significant positive correlation with R-Pb.con. The S-BCF_Pb_ exhibited a highly significant negative correlation with R-Pb.con, but a highly significant positive correlation with S-Pb.con. R-S+L-TF_Pb_ showed a highly significant negative correlation with R-Pb.con, while showing a highly significant positive correlation with S-Pb.con. S+L-G-TF_Pb_ showed a highly significant positive correlation with G-Pb.con. The S-BCF_Cd_ and G-BCF_Cd_ and R-S+L-TF_Cd_ showed a highly significant positive correlation with G-Cd.con, whereas S+L-G_TF_Cd_ showed no significant correlation with Cd content in the grains (**Figure 9D**).

## 4 Discussion

### 4.1 Gamma irradiation enhanced barley growth and barley grain yields under Cd and Pb stress

Ionizing radiation converts neutral atoms or molecules into reactive ions, triggering various biological effects and affecting plant signaling systems (Wang et al., 2022; De Francesco et al., 2023). Increasing evidence indicates that relatively low doses of gamma radiation can enhance plant growth under abiotic stress (Kang et al., 2024; Davood et al., 2022). In this study, the effects of gamma radiation on barley growth at different developmental stages under Cd and Pb stress were investigated. The results revealed that 35 and 50 Gy treatments promoted barley height during the tillering and jointing stages. Treatments of 35, 50, 120, and 150 Gy enhanced root length across all growth stages, while 50, 75, and 90 Gy increased the root-to-crown ratio throughout development. Notably, 50-Gy gamma irradiation exerted positive effects on all measured growth parameters at different stages under Cd and Pb stress. Furthermore, grain yield-related traits were positively influenced by gamma radiation doses ranging from 35 to 150 Gy under Cd and Pb stress. Sayed et al. (2025) reported that a low dose of gamma radiation (50 Gy) not only induces cytogenetic changes but also enhances drought tolerance and improves yield characteristics (Sayed et al., 2025). Kishore et al. (2024) demonstrated that appropriate doses of gamma radiation increase dragon fruit tolerance to abiotic stress (Kishore et al., 2024). Marzieh et al. (2022) found that optimum gamma radiation doses enhance grain yield, yield components, number of fertile florets, biological yield, plant height, harvest index, and flag leaf area (Marzieh et al., 2022). These findings are consistent with our results. The biopositive effects of low-dose gamma irradiation on plant growth under Cd and Pb stress may be attributed to increased synthesis of phytochelatins or secondary metabolites associated with stress tolerance during seedling development following seed irradiation.

### 4.2 The enrichment, absorption, and transport of Cd and Pb by barley are influenced by irradiation dose

Increasing evidence indicates that pre-exposure to gamma irradiation can enhance plant tolerance to various abiotic stressors, including heavy metal toxicity (Wang et al., 2017; Qi et al., 2015). However, the mechanisms by which low-dose gamma irradiation regulates heavy metal absorption and migration under Cd and Pb stress remain unclear. In this study, we investigated the accumulation and transport of Cd and Pb in different barley tissues at various growth stages under different gamma irradiation pretreatments. In this experiment, grain Pb content was reduced to the acceptable limit of 0.2 mg/kg under all irradiation treatments except 35 Gy. Although Cd content in barley grains exceeded the permissible limit of 0.1 mg/kg in all treatments, gamma irradiation reduced grain Cd content compared with the control (0 Gy). These differences may be attributed to two factors: (1) the enrichment and migration of Cd and Pb in the soil–barley system, and (2) the interaction mechanisms of Cd and Pb in the roots.

Heavy metals enter root cells from the soil solution and are transported to stems and leaves via the xylem by specific transporters. Root absorption, as well as the transport and redistribution of heavy metals within the roots, are critical factors determining the accumulation of heavy metals in edible plant parts (Uraguchi et al., 2009). The BCF represents the capacity of soil Cd or Pb to enter the plant, while the TF indicates the ability of roots to transfer Cd or Pb to grains. Both BCF and TF are key parameters for assessing the enrichment and migration of heavy metals in the soil–plant system (Meng et al., 2019; Munir et al., 2020). In this study, R-BCF_Cd_ values exceeded 1 under all treatments and growth stages, indicating a high capacity for Cd migration from soil to roots. In contrast, R-BCF_Pb_ values were less than 1 across all treatments and stages, likely due to competitive ion exchange between Pb and Cd ions (Wang et al., 2024). Numerous previous studies have reported that BCF and TF values for Cd are higher than those for Pb (Li et al., 2023; Arena et al., 2017). Additionally, BCF_Pb,_ BCF_Cd_ values in grains for both Pb and Cd were lower than those in roots, stems, and leaves, indicating that the migration and accumulation of heavy metals in barley grains were hindered. Compared with 0 Gy, all irradiation treatments increased BCF values of Pb and Cd in mature roots (except for 120 Gy in Cd), while decreasing BCF in the stems and grains (except for 35 Gy in Pb). Furthermore, R-S+L-TF_Cd_ (Cd translocation factor from root to stem-leaf), R-S+L-TF_Pb_ (Pb translocation factor from root to stem-leaf), S+L-G-TF_Cd_ (Cd translocation factor from stem-leaf to grain), and S+L-G-TF_Pb_ (Pb translocation factor from stem-leaf to grain) values were all below 1, with S+L-G-TF_Pb_ being particularly low (< 0.1), indicating that only a small fraction of heavy metals in aerial parts is transferred to the grains. All irradiation treatments reduced TF values of Pb and Cd across different tissues, except for 75 and 150 Gy in S+L-G-TF_Cd_ and 35 and 50 Gy in S+L-G-TF_Pb_. Furthermore, the Pearson correlation analysis showed that Cd content in the grains was significantly positively correlated with S- BCF_Cd_, G-BCF_Cd_, and R-S+L-TF_Cd_, while Pb content was significantly positively correlated with G-BCF_Pb,_ S+L-G-TF_Pb_. These results suggest that gamma irradiation can reduce heavy metal accumulation in barley grains by regulating the transport and accumulation of Cd and Pb within the plant.

## 5 Conclusions

The present study demonstrated that gamma irradiation of barley seeds can influence plant growth, yield-related parameters, and the accumulation and migration of Cd and Pb throughout the growth period. This resulted in increased the root length, root-shoot ratio, and yield components. Moreover, doses ranging from 50 to 150 Gy reduced Cd and Pb contents in the grains by increasing their accumulation in roots and inhibiting their absorption and transport within the soil–plant system. Gamma irradiation should be viewed as a component of an integrated strategy, potentially combined with soil amendments, phytoremediation cycles, or the cultivation of low-accumulating crop varieties to achieve compliance with safety standards.

## Data Availability

The original contributions presented in the study are included in the article/supplementary material. Further inquiries can be directed to the corresponding author/s.
